# A Spike-Linked HPV16 E7 DNA Vaccine Induces Potent Antitumor and Anti-Spike Immune Responses

**DOI:** 10.3390/ijms27146249

**Published:** 2026-07-14

**Authors:** Yichu Xu, Yining Liu, Yu-Cheng Chang, Ya-Chea Tsai, Chuan-Hsiang Huang, Tzyy-Choou Wu, Chien-Fu Hung

**Affiliations:** 1 Department of Pathology, Johns Hopkins University School of Medicine, 1550 Orleans Street, CRB II 307, Baltimore, MD 21287, USA; yxu209@alumni.jh.edu (Y.X.); chuang29@jhmi.edu (C.-H.H.); 2 Department of Biochemistry and Molecular Biology, Johns Hopkins University Bloomberg School of Public Health, Baltimore, MD 21287, USA; 3 Department of Cell Biology, Johns Hopkins University, Baltimore, MD 21287, USA; 4 Department of Obstetrics and Gynecology, Johns Hopkins University School of Medicine, Baltimore, MD 21287, USA; 5 Department of Molecular Microbiology and Immunology, Johns Hopkins University School of Medicine, Baltimore, MD 21287, USA; 6 Department of Oncology, Johns Hopkins University School of Medicine, Baltimore, MD 21287, USA

**Keywords:** DNA vaccine, spike protein, cancer, immunotherapy

## Abstract

Persistent infection with high-risk human papillomavirus (HPV), particularly HPV16, is a major driver of HPV-associated cancers; however, strategies for treating established HPV-induced tumors remain scarce. Here, we developed a DNA-based vaccine linking the SARS-CoV-2 spike (S) protein with an HPV16 E7 epitope (aa 49-57) to simultaneously induce antiviral humoral immunity and antitumor cellular responses. We generated 2 constructs, S-E7 and S-RE7, with the latter incorporating a furin cleavage site (R) to enhance antigen processing. In vitro, S-RE7 significantly enhanced E7-specific CD8^+^ T cell activation compared to S-E7, highlighting the importance of the furin sequence. In vivo, both S-linked vaccines elicited robust E7-specific CD8^+^ T cell responses and provided complete protection against TC-1 tumor challenge in a prophylactic murine model, with long-lasting immunity upon tumor rechallenge. In therapeutic settings, vaccination with S-E7 or S-RE7 significantly suppressed tumor growth, extended survival, and reduced circulating myeloid-derived suppressor cells (MDSCs), indicating alleviation of systemic immunosuppression. Notably, S-RE7 demonstrated faster antitumor effects overall in early tumor progression. In addition to cellular immunity, both constructs induced high levels of anti-spike antibodies, with S-RE7 eliciting approximately fourfold higher responses than S-E7. Furthermore, S-RE7 effectively boosted pre-existing anti-spike immunity in mice that were previously vaccinated. This “two-in-one” strategy represents a promising and versatile platform for the prevention and treatment of HPV-associated cancers while maintaining preparedness against potential SARS-CoV-2.

## 1. Introduction

High-risk human papillomavirus (HPV) can be carcinogenic under conditions of persistent infection, resulting in HPV-associated cancers such as cervical, penile, vaginal, anal, and oropharyngeal cancers [1,2]. Among the high-risk genotypes, HPV16 is the predominant type responsible for the majority of HPV-associated cancers [3]. The oncogenic potential of high-risk HPV is mediated by the viral oncoproteins E6 and E7, which promote malignant transformation through degradation of the tumor suppressors p53 and pRb, respectively [4,5]. Prophylactic vaccines such as Gardasil-9 and Cervarix have substantially reduced the incidence of HPV infections [6]; however, they provide no therapeutic benefit to individuals with existing HPV infections because these vaccines primarily induce humoral responses rather than T cell-mediated cellular immunity [7,8]. Consequently, numerous therapeutic HPV vaccines have been tested in preclinical and clinical studies, but none are currently approved [9]. Thus, there is an urgent need to develop novel immunotherapeutic approaches for HPV-associated malignancies.

Among clinical trials, DNA-based vaccines, such as VGX-3100 and GX-188E, have demonstrated more consistent efficacy compared with protein- or vector-based vaccines [10,11]. DNA vaccines offer several advantages: they are effective in inducing both humoral and cellular immunity, stable and resistant to temperature fluctuations, and cost-effective to manufacture and store. Furthermore, DNA plasmids can be designed to encode multiple epitopes or antigens, making them a versatile platform for developing broadly protective, multivalent vaccines against various strains or pathogens [12]. DNA vaccines encoding antigen alone exhibit low immunogenicity, largely due to limited cellular and nuclear uptake of the plasmid DNA [13]. Therefore, in addition to technical strategies that improve outcomes, such as electroporation, high-dose administration, and booster immunizations, complementary approaches including molecular adjuvants or strategies that enhance antigen presentation are often required to strengthen immune responses [14,15].

Severe acute respiratory syndrome coronavirus 2 (SARS-CoV-2), the causative agent of the 2019 global pandemic, has been extensively studied since its emergence. Viral entry is mediated by the trimeric spike (S) glycoprotein, which is highly immunogenic and capable of eliciting robust humoral and cellular immune responses [16,17]. Zhang et al. reported that fusion of the heptad repeat 1 (HR1) and HR2 sequences from SARS-CoV-2 S2 subunit to HPV-16 E6/E7 in adenovirus vector enhanced E6/E7-specific CD8^+^ T cell-based immune responses and improved antitumor efficacy in both prophylactic and therapeutic models [18]. They reasoned this immune enhancement is associated with the self-assembling (trimerization) properties of the HR domains [19]. Based on these findings, we designed a DNA-based vaccine encoding the HPV16 E7 epitope (aa 49-57) fused to the C-terminus of full-length S (hereafter referred to as S-E7) and hypothesized that this construct would elicit robust humoral responses against SARS-CoV-2 as well as potent cellular immunity against HPV-associated cancers.

To further enhance vaccine efficacy, we also designed a variant of the S-E7 construct incorporating a furin cleavage sequence arginine-valine-lysine-arginine (R-V-K-R) between the full-length S and E7, hereafter referred to as S-RE7. Furin is a serine endoprotease predominantly localized in the trans-Golgi network (TGN) that recognizes and cleaves substrates containing the canonical multibasic consensus sequence R-X-K/R-R [20,21,22,23]. Incorporation of a furin cleavage site between antigens or cytotoxic T lymphocyte epitopes has been shown to enhance epitope processing and presentation on major histocompatibility complex class I (MHC I) molecules through a transporter associated with antigen processing (TAP)-independent pathway [24,25,26]. This strategy has been well-characterized and widely adopted in nucleic acid-based vaccine design to improve immunogenicity, with several constructs advancing to clinical evaluation [27,28,29,30].

Our vaccine design is a “kill two birds with one stone” approach that allows the vaccine to provide immunity against SARS-CoV-2 in case of viral recurrence while simultaneously preventing and treating HPV-associated cancers.

## 2. Results

### 2.1. Construction of Spike-Linked Vaccines

**Figure 1:**
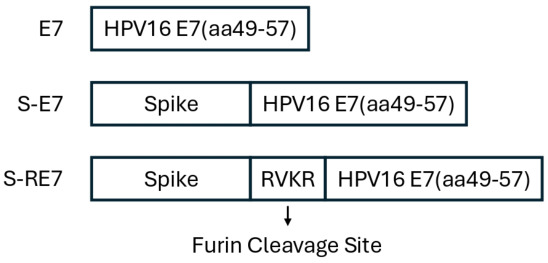
DNA construct of pcDNA3-E7(49-57), pcDNA3-S-E7(49-57), and pcDNA3-S-RE7(49-57). The HPV16 E7 CTL epitope (aa49-57) was cloned into the pcDNA3 vector to produce the pcDNA3-E7(49-57). The HPV16 E7 epitope (aa49-57), with or without a furin cleavage site, was fused to the C-terminus of the full-length spike (S) protein and cloned into the pcDNA3 vector to generate the fusion constructs pcDNA3-S-RE7(49-57) and pcDNA3-S-E7(49-57).

The S-E7 sequence was generated by fusing the HPV16 E7 epitope (aa 49-57) to the C-terminus of the full-length SARS-CoV-2 S protein. The S-RE7 sequence was constructed similarly, but with an additional furin cleavage site (arginine-valine-lysine-arginine) inserted between the S protein and the E7 epitope. A construct encoding the HPV16 E7 epitope alone was also generated (Figure 1). Each sequence was individually cloned into the pcDNA3 vector to generate the plasmids pcDNA3-S-E7, pcDNA3-S-RE7, and pcDNA3-E7. These plasmids were used as DNA vaccines for immunization.

### 2.2. Furin Cleavage Sequence Enhances the Immunogenicity of Spike-Linked Vaccine in Vitro

**Figure 2:**
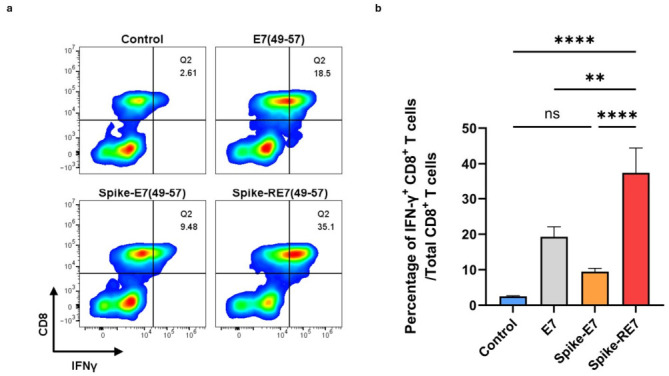
S-RE7(49-57) induces robust T cell activation in vitro. DC2.4 were transfected with 1 ug of (1) pcDNA3 vector, (2) pcDNA3-E7(49-57), (3) pcDNA3-S-E7(49-57), or (4) pcDNA3-S-RE7(49-57), and then co-cultured with HPV16 E7 aa49-57 peptide-specific CD8^+^ T cells. Following incubation, the E7-specific T cells were stained for surface CD8 and intracellular IFN-γ, then analyzed by flow cytometry. (**a**) Representative flow cytometry pseudocolor plot illustrating the proportion of activated E7-specific CD8^+^ T cells. Color indicates event density, with blue representing the lowest density and red representing the highest density of events. (**b**) Bar graph summary of the flow cytometry results. Data are presented as mean ± SD of three biological replicates (**a**) and analyzed using one-way ANOVA with a Tukey post hoc test (**b**). ** *p* < 0.01, **** *p* < 0.0001, ns: not significant.

To evaluate the immunogenicity of these constructs, we first performed an in vitro intracellular interferon (IFN)-γ assay. Dendritic cells were transfected with the indicated constructs and then co-cultured with E7-specific CD8^+^ T cells. Flow cytometry analysis showed that the S-RE7 construct induced a significantly higher proportion of activated E7-specific CD8^+^ T cells (Figure 2a,b). In contrast, the S-E7 construct showed no significant difference compared with the control group, indicating the importance of incorporating a furin cleavage site in the vaccine design.

### 2.3. Spike-Linked Vaccines Have Prophylactic Efficacy in TC-1 Mice Models

**Figure 3:**
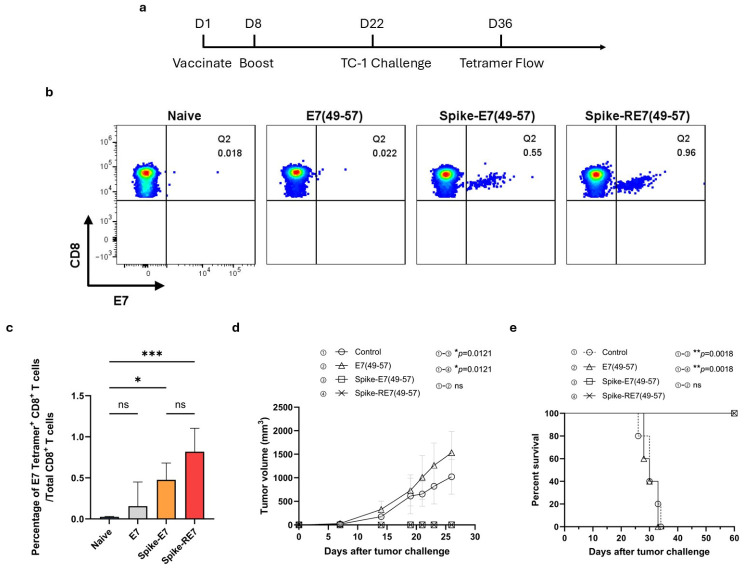
Two-dose immunization of S-linked vaccines generates protection in a prophylactic model. (**a**) Timeline of the experiment. On day 1, mice (*n* = 5 per group) were immunized with (1) 2 μg of pcDNA3-E7(49-57), (2) 2 μg of pcDNA3-S-E7(49-57), or (3) 2 μg of pcDNA3-S-RE7(49-57) via intramuscular electroporation. On day 8, the mice were boosted using the same regimen. On day 22, mice were subcutaneously challenged with 1 × 10^5^ TC-1 tumor cells. Tumor size and survival were subsequently monitored. On day 36, PBMCs were collected, and lymphocytes were analyzed via flow cytometry. (**b**) Representative flow cytometry pseudocolor plot showing the median frequency of E7-specific CD8^+^ T cells within the CD8^+^ T cell population. Color indicates event density, with blue representing the lowest density and red representing the highest density of events. (**c**) Bar graph summarizing the quantitative results of the flow cytometry analysis. (**d**) Line graph illustrating tumor volume progression in TC-1 tumor-challenged mice. (**e**) Kaplan–Meier analysis was used to assess the 60-day survival of mice bearing TC-1 tumors. Data are presented as mean ± SD and analyzed by one-way ANOVA with a Tukey post hoc test (**c**), repeated measures ANOVA followed by a Tukey post hoc test (**d**), and the Kaplan–Meier method with log-rank (Mantel–Cox) test (**e**). * *p* < 0.05, ** *p* < 0.01, *** *p* < 0.001, ns: not significant.

To evaluate the antitumor activity of these constructs, we employed the TC-1 prophylactic murine tumor model, a widely used HPV16 E6/E7-expressing system for evaluating E7-specific antitumor immunity. Mice were vaccinated intramuscularly in the hind leg with E7, S-E7, or S-RE7, followed by electroporation. A second dose was given one week later using the same regimen. A one-week prime-boost interval was selected based on established vaccination protocols commonly used in T-cell immunology to induce robust antigen-specific immune responses. Two weeks after the final vaccination, mice were subcutaneously challenged with 1 × 10^5^ TC-1 tumor cells, with unvaccinated tumor-challenged mice serving as naïve controls. Two weeks after tumor challenge, peripheral blood mononuclear cells (PBMCs) were collected and analyzed by flow cytometry to evaluate antigen-specific CD8^+^ T-cell responses (Figure 3a). E7-specific CD8^+^ T cells were quantified using E7 peptide-loaded MHC class I tetramer staining, a well-established surrogate for functional cytotoxic T lymphocyte activation [31]. Mice vaccinated with either S-E7 or S-RE7 exhibited robust E7-specific CD8^+^ T cell responses (Figure 3b,c). Tumor growth and survival were monitored. Notably, mice receiving either S-E7 or S-RE7 vaccination showed no detectable tumor growth and survived throughout the study (Figure 3d). Interestingly, in contrast to the in vitro results, the vaccine lacking the furin cleavage site remained effective in vivo, as no significant difference was observed between the two S-linked vaccine groups. Together, these findings demonstrate that the S-linked vaccines can induce potent antigen-specific immune responses and provide effective protection against HPV-associated cancer in vivo.

### 2.4. Spike-Linked Vaccines Offer Durable Protection

**Figure 4:**
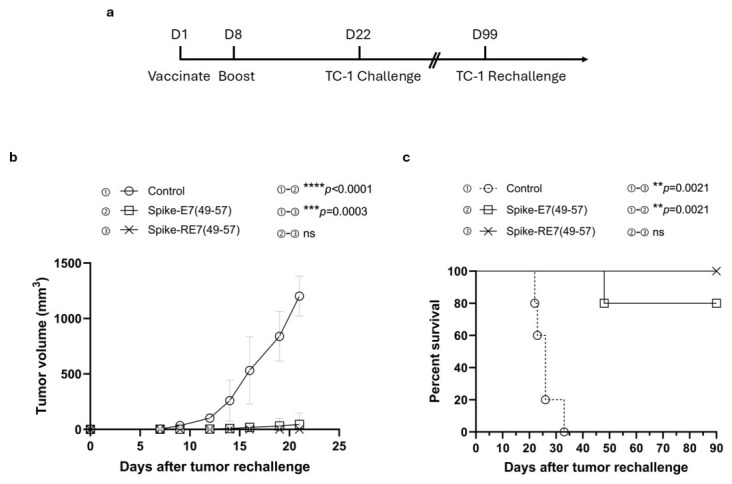
Two-dose immunization of S-linked vaccines elicits long-lasting protection. (**a**) Timeline of the experiment. On day 1, mice (*n* = 5 per group) were immunized with (1) 2 μg of pcDNA3-E7(49-57), (2) 2 μg of pcDNA3-S-E7(49-57), or (3) 2 μg of pcDNA3-S-RE7(49-57) via intramuscular electroporation. On day 8, the mice were boosted using the same regimen. On day 22, mice were subcutaneously challenged with 1 × 10^5^ TC-1 tumor cells. Sixty days after the initial tumor challenge, mice were rechallenged with 1 × 10^5^ TC-1 tumor cells. Tumor size and survival were monitored. (**b**) Line graph showing tumor volume progression in TC-1 tumor-rechallenged mice. (**c**) Kaplan–Meier analysis was used to assess the 60-day survival of mice bearing rechallenged TC-1 tumors. Data are presented as mean ± SD and analyzed using repeated measures ANOVA followed by a Tukey post hoc test (**b**), and the Kaplan–Meier method with log-rank (Mantel–Cox) test (**c**). ** *p* < 0.01, *** *p* < 0.001, **** *p* < 0.0001, ns: not significant.

To evaluate the durability of the vaccine-induced antitumor response, mice were rechallenged with TC-1 tumor 7 weeks after the initial challenge using the same regimen. Mice vaccinated with S-RE7 remained tumor-free for the duration of the experiment, whereas one mouse in the S-E7 group succumbed to tumor burden (Figure 4). However, no statistical significance was observed between the two vaccine groups. These results indicate that the S-linked vaccines provide durable and effective tumor protection in vivo.

### 2.5. Spike-Linked Vaccines Show Therapeutic Potential in Tumor-Bearing Mice

**Figure 5:**
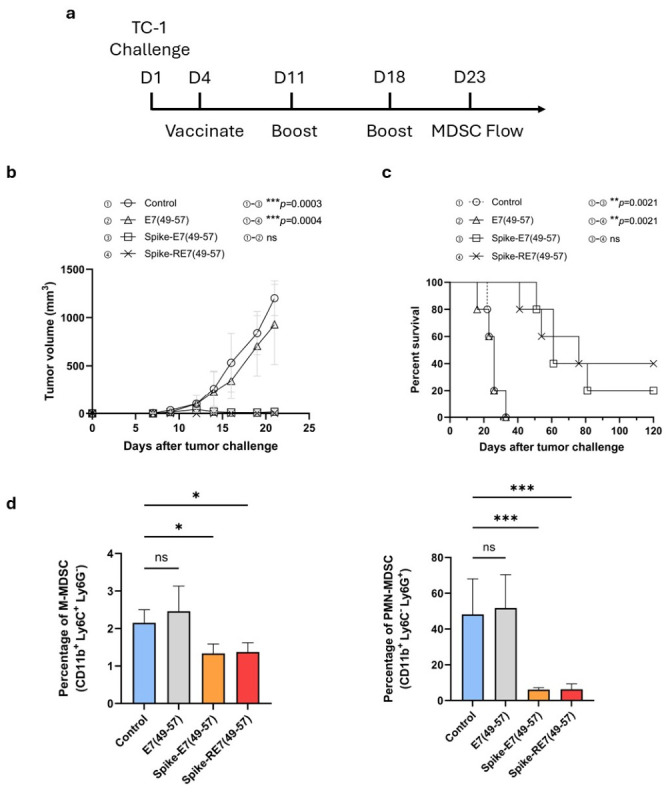
S-linked vaccines suppress tumor growth and extend survival in a therapeutic model. (**a**) Timeline of the experiment. On day 1, mice (*n* = 5 per group) were subcutaneously challenged with 1 × 10^5^ TC-1 tumor cells. On day 4, mice were immunized intramuscularly with (1) 10 μg of pcDNA3-CRT-E7(49-57), (2) 10 μg of pcDNA3-S-E7(49-57) or (3) 10 μg of pcDNA3-S-RE7(49-57), followed by electroporation. Booster immunizations using the same regimen were administered on days 11 and 18. Tumor size and survival were subsequently monitored. On day 23, PBMCs were collected, and lymphocytes were analyzed via flow cytometry. (**b**) Line graph illustrating tumor volume progression in TC-1 tumor-challenged mice. (**c**) Kaplan–Meier analysis assessing 120-day survival of mice bearing TC-1 tumors. (**d**) Bar graphs summarizing the flow cytometry analysis of M-MDSCs and PMN-MDSCs. Data are presented as mean ± SD and analyzed by two-way ANOVA with mixed-effect model followed by a Tukey post hoc test (**b**), Kaplan–Meier method with log-rank (Mantel–Cox) test (**c**), and one-way ANOVA with Tukey post hoc test (**d**). * *p* < 0.05, ** *p* < 0.01, *** *p* < 0.001, ns: not significant.

Based on the findings that S-linked vaccines could prophylactically protect mice against TC-1 tumor, we next investigated the therapeutic potential of the vaccines in mice bearing TC-1 tumors. Mice were subcutaneously challenged with TC-1 tumor cells, followed by leg intramuscular administration of S-E7, S-RE7, or E7 after three days. Two booster doses were administered weekly after the initial vaccination using the same regimen (Figure 5a). This vaccination schedule was chosen to allow adequate tumor establishment while providing sufficient time for the induction and expansion of vaccine-induced T-cell responses. Mice treated with S-linked vaccines developed significantly smaller tumors and had prolonged survival compared to both the untreated control group and the group receiving the E7 (Figure 5b). Notably, in the 15-day window after tumor challenge, mice given S-RE7 remained tumor-free whereas mice given S-E7 had increased tumor volume until day 12 and then the progression was suppressed between day 12 and day 15, suggesting that having a furin cleavage site might be helpful to make the vaccine take effectiveness faster (Figure S1). Moreover, vaccination lowered both circulating granulocytic myeloid-derived suppressor cells (PMN-MDSCs) and monocytic MDSCs (M-MDSCs), indicating reduced systemic immunosuppression (Figure 5c). These results highlight that S-linked vaccines could serve as an effective therapeutic strategy for controlling tumor progression and improving survival.

### 2.6. Spike-Linked Vaccines Generate High Levels of Anti-Spike Antibodies

**Figure 6:**
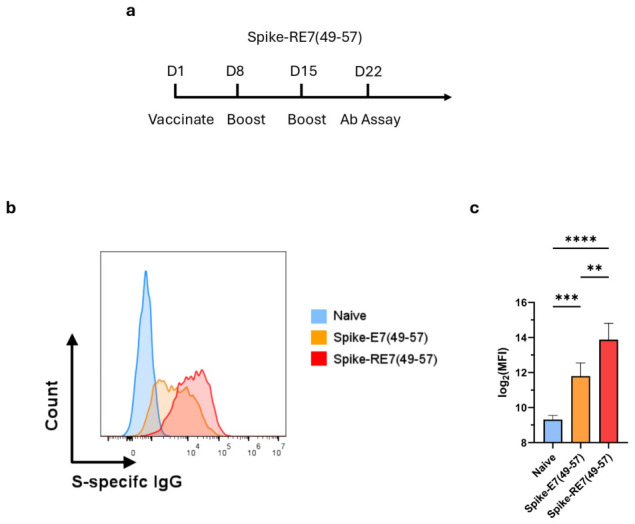
S-RE7(49-57) increases serum anti-spike IgG levels. (**a**) Timeline of the experiment. On day 1, mice (*n* = 5 per group) were immunized with (1) 10 μg of pcDNA3-E7(49-57) or 10 μg of pcDNA3-S-RE7(49-57) via intramuscular electroporation. On days 8 and 15, the mice were boosted using the same regimen. On day 22, sera were collected and anti-spike antibody levels were analyzed by flow cytometry. (**b**) Representative staggered histogram illustrating spike-specific IgG levels in mice. (**c**) Bar graph summarizing flow cytometry results, with the *y*-axis representing the log_2_ of median fluorescence intensity (MFI). Data are presented as mean ± SD and analyzed using one-way ANOVA with Tukey post test (**c**). ** *p* < 0.01, *** *p* < 0.001, **** *p* < 0.0001.

Since the vaccines encode full-length S protein, the translated protein is expected to follow the secretory pathway and be expressed on the cell surface, mimicking wild-type S protein and enabling B cell-mediated immune activation. To assess the humoral response, mice were vaccinated with S-E7 or S-RE7 and received two weekly booster doses using the same regimen. One week after the final immunization, sera were collected and evaluated for anti-spike antibody levels (Figure 6a). HeLa cells were transfected with SARS-CoV-2-S plasmid to ensure comparable surface expression of S protein and then incubated with sera from vaccinated mice followed by fluorescent anti-IgG. Flow cytometry analysis revealed that both S-linked constructs elicited significantly higher anti-spike antibodies compared with control sera. Notably, at a 1:500 dilution, the median fluorescence intensity for S-RE7 sera was approximately fourfold higher than S-E7 (Figure 6b,c). Thus, the data demonstrate that our S-linked vaccines are capable of inducing robust humoral immune response.

**Figure 7:**
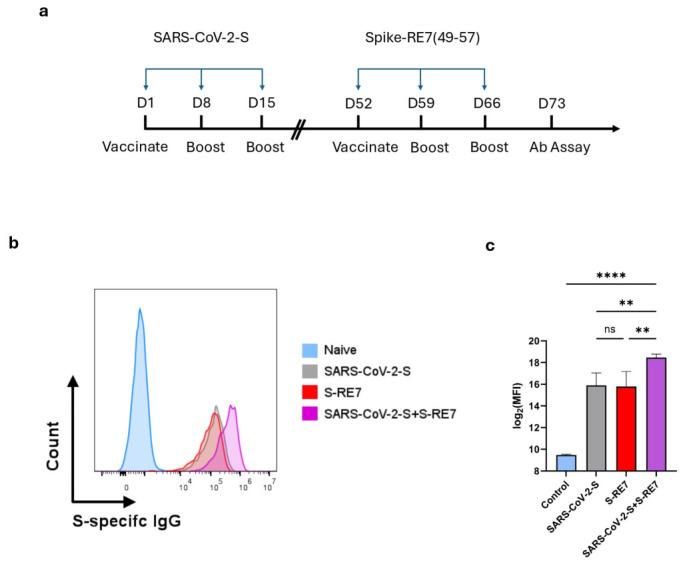
S-RE7(49-57) induces comparable immune responses to the SARS-CoV-2-S DNA vaccine and acts as a booster. (**a**) Timeline of the experiment. On day 1, mice (*n* = 5 per group) were immunized intramuscularly with 10 μg of pCMV3-SARS-CoV-2-S, followed by electroporation. On days 8 and 15, the mice were boosted using the same regimen. On day 52, mice were immunized with 10 μg of pcDNA3-S-RE7(49-57) via intramuscular electroporation. On days 59 and 66, the mice (*n* = 5) were boosted using the same regimen. On day 73, sera were collected from mice and anti-spike antibody levels were analyzed by flow cytometry. (**b**) Representative staggered histogram illustrating spike-specific IgG levels in mice. (**c**) Bar graph summarizing flow cytometry results, with the *y*-axis representing the log_2_ of median fluorescence intensity (MFI). Data are presented as mean ± SD and analyzed using one-way ANOVA with Tukey post test (**c**). ** *p* < 0.01, **** *p* < 0.0001, ns: not significant.

Given the widespread pre-existing immunity to SARS-CoV-2, we evaluated whether our S-linked vaccines could function as a booster in a murine model. Based on its enhanced therapeutic efficacy against established tumor and robust induction of humoral responses, S-RE7 was chosen for subsequent experiments. Mice were initially vaccinated with SARS-CoV-2-S, followed by two weekly booster doses using the same regimen (Figure 7a). One month after the final immunization, sera were collected and analyzed for anti-spike antibody levels. Flow cytometry showed a significant increase in anti-spike antibodies in vaccinated mice, confirming successful immunization (Figure S2). One week later, to assess S-RE7 as a booster, the same vaccination regimen used for evaluating the humoral responses of S-linked vaccines was applied (Figure 7a). Sera were collected a second time from mice previously vaccinated with SARS-CoV-2-S. As shown in Figure 7b,c, co-administration of SARS-CoV-2-S and S-RE7 elicited significantly higher anti-spike antibody levels than either vaccine alone. Consistent with earlier findings, S-RE7 vaccination induced a marked increase in anti-spike antibody levels relative to controls.

## 3. Discussion

HPV infection accounts for 5% of cancers worldwide, with an estimated 700,000 new HPV-associated cancer cases each year [32]. In addition to conventional treatment options such as surgery, radiotherapy, and chemotherapy, immunotherapy has emerged as a promising strategy for the treatment of HPV-associated malignancies [33]. Currently, a multidisciplinary treatment approach is often required to effectively manage these cancers. Although these therapeutic options have demonstrated clinical benefits, several challenges remain, including inconsistent treatment outcomes [34], drug resistance [35,36], and treatment-related toxicity [37]. Therefore, considerable research effort has focused on developing novel therapeutic vaccines for HPV-associated cancers to reduce disease incidence and improve patient outcomes. In this study, we designed two S-linked DNA vaccines, S-E7 and S-RE7, by incorporating the full-length SARS-CoV-2 S protein and the HPV-16 E7 epitope (aa 49-57), with or without the furin cleavage sequence, respectively. Our results demonstrated that these vaccines elicited robust antitumor activity through the induction of strong CD8^+^ T-cell responses and the reduction in circulating MDSCs. Notably, the vaccines also generated potent humoral immune responses against SARS-CoV-2. These findings suggest that the S-linked DNA vaccine platform represents a promising therapeutic strategy for the treatment of HPV-associated cancers and additionally offers potential protection against future coronavirus outbreaks.

The rationale for linking the HPV16 E7 epitope to the C-terminus of the S protein was to preserve the native conformation of the receptor-binding domain (RBD), which is proximal to the N-terminus and is the site of binding for most neutralizing antibodies [38,39,40].

The S protein is inherently immunogenic and is known to induce pro-inflammatory cytokines such as IFN-γ [41]; we hypothesized that fusing it with the E7 epitope would enhance antigen-specific immunogenicity. Based on our extensive previous experience with TC-1 tumor models and findings from other studies, antitumor immunity is almost always dependent on CD8^+^ T cells [42,43,44]. Therefore, we focused on characterizing E7-specific CD8^+^ T cell responses after vaccination. Notably, anti-E7 humoral responses do not mediate tumor clearance in this model, as HPV E7 resides intracellularly and cannot be neutralized by circulating antibodies.

We observed that S-E7 transfection enhanced E7-specific CD8^+^ T cell activation in vitro, leading us to conduct in vivo studies to assess the efficacy of the vaccines. In prophylactic studies, both S-linked vaccines demonstrated potent efficacy, providing complete protection against TC-1 tumor challenge and eliciting robust E7-specific T cell responses. Following tumor rechallenge, all mice that received S-RE7 vaccination remained tumor-free. Notably, although one mouse in the S-E7 group died due to tumor burden, overall survival did not differ significantly between the two vaccine groups. For therapeutic studies, we increased the vaccine dose and introduced an additional booster to counter the aggressive nature of the TC-1 tumor model. Both S-linked vaccines markedly reduced tumor progression, extended survival, and resulted in decreased circulating MDSC levels. Overall, these findings highlight the potential of S-linked vaccines in preventing and treating HPV-associated malignancies.

To assess the ability of these vaccines to induce humoral immunity against SARS-CoV-2, we quantified serum anti-spike antibody levels. Both S-linked vaccines significantly increased antibody production, with S-RE7 inducing approximately fourfold higher levels than S-E7. Given the stronger humoral response elicited by S-RE7, we evaluated its capacity to enhance pre-existing immunity. As expected, S-RE7 further augmented anti-spike antibody levels in mice previously immunized against SARS-CoV-2, supporting its potential as a “booster” candidate. Although direct viral challenge was not performed, these results suggest that S-linked vaccines could potentially provide protection against future SARS-CoV-2 exposure.

DNA vaccination elicits cellular immune responses primarily through cross-presentation, which is a complex process involving multiple intracellular pathways [45,46,47]. Given that the wild-type trimeric S protein is formed in the endoplasmic reticulum (ER) and transported to the plasma membrane via the conventional secretory pathway [17,48], we hypothesized that our S-linked constructs (S-E7 and S-RE7) would follow the same biosynthetic route. In the context of cross-presentation in vivo, DCs can acquire E7-linked S protein released from plasmid-transfected cells. For the vacuolar pathway, internalized exogenous antigens are processed by endosomal proteases, and the resulting epitopes are loaded directly onto recycling MHCI molecules within the endosome [49]. This process is TAP-independent and results in the subsequent trafficking of peptide-loaded complexes to the plasma membrane. In this case, the enhanced therapeutic benefit observed with S-RE7 in murine models could be attributed to the presence of the furin cleavage site. Furin is known to traffic dynamically between the endosome and TGN as part of its normal itinerary [50]. Thus, the furin site may facilitate antigen processing in the vacuolar pathway, potentially accelerating the onset of therapeutic effects. Interestingly, we observed that S-E7 had a notable discrepancy between in vitro and in vivo results. The S-E7 construct elicited weak CD8^+^ T cell activation in transfected DC cultures, yet it retained therapeutic efficacy against HPV-associated cancers in murine models. This divergence can be explained by the topology and trafficking of the fusion S protein. In both S-E7 and S-RE7, the E7 epitope is fused to the C-terminus (cytoplasmic tail) of the S protein, which theoretically orients toward the cytosol [51,52]. For S-E7, it is possible that the fusion protein adopts a stable conformation since the length of the epitope is considerably shorter than the full-length S protein. Thus, the E7-linked S protein could traffic through the secretory pathway similarly to wild-type S protein, resulting in little proteasomal degradation and minimal generation of epitope for direct MHC class I presentation in vitro. The therapeutic effect observed in vivo likely results from cross-presentation. In contrast, transfection with S-RE7 in vitro resulted in robust T cell activation. One possible explanation is that the highly charged nature of the furin cleavage sequence may cause suboptimal folding of the S protein. These aberrant proteins would then be selected by molecular chaperones and targeted to the ER-associated degradation machinery [53], liberating the E7 epitope for MHC class I presentation and thereby activating more E7-specific cytotoxic T cells. In vivo, proteasome-mediated degradation of the S-RE7 may generate increased amounts of S-derived epitopes, which can be taken up by DCs and presented on MHCII molecules, leading to enhanced humoral immunity compared to S-E7. In summary, while furin-mediated cleavage of the E7 epitope is unlikely to occur in our in vitro T cell activation assay, the presence of the furin cleavage site may play a role in facilitating antigen processing during cross-presentation in vivo.

Immunotherapy plays a central role in the treatment of cancer; however, tumors can develop resistance through a variety of mechanisms. This challenge is evident in HPV-associated malignancies, which remain a significant global health concern. While S-linked vaccines exhibit excellent treatment outcomes, combining them with other therapeutic agents may be necessary to overcome resistance. In the present study, we utilized a single HPV16 E7 epitope (aa 49-57) to establish proof of concept for the S-linked vaccine platform. Incorporation of multiple CTL epitopes or neoantigens could broaden immune coverage, reduce immune escape, and improve therapeutic efficacy [54]. Thus, our S-linked vaccines provide a versatile platform for the development of next-generation DNA vaccines for treating diverse cancer types. It is worth mentioning that the antitumor efficacy of this platform in hosts with pre-existing anti-spike immunity remains an important unresolved clinically relevant question. Further dedicated research is needed to elucidate how prior S protein immunization influences vaccine-mediated tumor protection.

Despite the encouraging outcomes, our study has several limitations. First, the precise role of the furin cleavage site in our vaccine design remains unelucidated. While we proposed plausible mechanistic speculations, further investigation is required. Second, detailed characterization of memory T-cell differentiation was not performed. Although durable therapeutic efficacy was observed, further analysis of memory subsets, including central memory and effector memory T cells, would provide additional insight into the vaccine-induced long-term immune response. Third, circulating MDSC levels may be a good indicator of overall tumor progression, but they do not capture the immunosuppressive characteristics within the tumor microenvironment [55]. Evaluating tumor-infiltrating MDSCs would be insightful in providing a more comprehensive immunological picture. Fourth, we employed a subcutaneous tumor model for simplicity; however, orthotopic models would better reflect organ-specific microenvironments and therapeutic responses. Lastly, while DNA vaccines delivered by electroporation have been extensively evaluated in preclinical and clinical studies and generally demonstrate a favorable safety profile with no vaccine-associated severe adverse effects reported [51,52], the translation of DNA vaccines from murine models to clinical settings remains challenging due to differences between murine and human immune systems, as well as stringent regulatory guidelines regarding the potential risk of genomic integration and the delivery system [12,56].

## 4. Methods

### 4.1. Plasmid DNA Constructs and Preparation

The generation of pcDNA3-E7(49-57) [57] and pCMV3-SARS-CoV-2-S, which contains DNA encoding the S protein from the Wuhan variant of SARS-CoV-2 [58], has been described previously. To generate pcDNA3-S-E7(49-57), encoding the Wuhan S protein and the HPV16 E7 amino acid (aa) 49-57 epitope (RAHYNIVTF), the sequence was synthesized by GenScript Corporation and cloned into the EcoRI and ApaI restriction sites of pcDNA3.1(-). Similarly, pcDNA3-S-RE7(49-57), which encodes the Wuhan S protein, the furin cleavage site RVKR, and the HPV16 E7 aa 49-57 epitope (RVKRRAHYNIVTF), was synthesized by GenScript and cloned into the same restriction sites of pcDNA3.1(-). Plasmid DNA was prepared using the HiSpeed Plasmid Maxi Kit (QIAGEN, Valencia, CA, USA), with purity assessed by an A260/A280 ratio of approximately 1.8.

### 4.2. Cell Lines

HPV16 E6- and E7-expressing TC-1 cells were generated by us [27] and DC2.4 dendritic cells were provided by Dr. Kenneth Rock from the Department of Pathology at Harvard Medical School [28,29] as previously described. Both cell lines were maintained in RPMI medium supplemented with 2 mM glutamine, 1 mM sodium pyruvate, 100 IU/mL penicillin, 100 μg/mL streptomycin, and 10% fetal bovine serum (FBS). The murine HPV16 E7 peptide (aa 49-57)-specific CD8^+^ T cell line was established as previously described [59]. Briefly, E7-specific CD8^+^ splenocytes were isolated from mice vaccinated with E7 DNA and co-cultured with E7-expressing target cells. To enrich E7-specific CD8^+^ T cells, the cells were maintained with repeated antigen stimulation under conditions supporting antigen-specific expansion. Spike-expressing cells were generated by transiently transfecting 293T cells with spike plasmids using Lipofectamine 2000 (ThermoFisher, Waltham, MA, USA).

### 4.3. Mice

Female C57BL/6 mice aged 6-8 weeks were purchased from Charles Rivers Laboratories (Frederick, MD, USA) and housed under specific pathogen-free conditions at the Johns Hopkins University School of Medicine Animal Facility (Baltimore, MD, USA). All animal procedures were performed in accordance with protocols approved by the Johns Hopkins Institutional Animal Care and Use Committee (IACUC; protocol MO23M84, approved on 20 March 2023). All experiments were conducted in compliance with institutional and national guidelines for the care and use of laboratory animals.

### 4.4. In Vitro T Cell Activation Assay

To assess the ability of the plasmid construct to activate CD8^+^ T cells, DC2.4 cells were transfected with 1 μg of one of the following constructs: (1) empty pcDNA3 vector, (2) pcDNA3-E7, (3) pcDNA3-S-E7(49-57), or (4) pcDNA3-S-RE7(49-57). After 24 h, 2.5 × 10^5^ transfected cells were collected and co-cultured with an equal number of HPV16 E7 (amino acids 49-57)-specific CD8^+^ T cells in 96-well round-bottom plates. A total of 10 μg/mL of GolgiPlug (BD Pharmingen, San Diego, CA, USA) was added to the culture for a period of 20–24 h. T cells were then stained with Zombie Aqua viability dye (Fixable Viability Kit; Biolegend, San Diego, CA, USA) to discriminate live and dead cells, followed by Fc Block (Biolegend) treatment to reduce nonspecific antibody binding. After washing, cells were fixed and permeabilized using Perm/Fix solution (Invitrogen, Thermo Fisher Scientific, Waltham, USA), then stained with APC-A750-conjugated anti-mouse CD8α antibody (1:1000) and FITC-conjugated anti-mouse intracellular IFN-γ antibody (1:500; Biolegend) for 30 min at 4 °C. Samples were analyzed on CytoFLEX S (Beckman Coulter, Brea, CA, USA)to assess HPV16 E7-specific CD8^+^ T cell activation.

### 4.5. DNA Vaccination

For intramuscular vaccination, plasmids were prepared and injected using a 31-gauge needle at the indicated dose into the tibialis muscle of the shaved hind legs of mice, followed by electroporation using an ECM830 Square Wave Electroporation System (BTX Harvard Apparatus, Holliston, MA, USA). Each leg received half of the total vaccine dose in 30 µL of PBS. The electroporator was set to deliver 8 pulses at 106 V with a 20 ms pulse duration and 200 ms interval. Booster vaccination(s) were administered using the same dose and regimen as the priming vaccination.

### 4.6. Detection of E7-Specific CD8^+^ T Cells and MDSCs

Blood samples were obtained from mice through submandibular puncture and collected into tubes containing 100 µL of EDTA (0.5 M, pH 8). PBMCs were prepared by lysing red blood cells with RBC lysis buffer (ThermoFisher, Waltham, MA, USA) followed by filtration through a cell strainer plate. Prior to antibody labeling, cells were incubated with Zombie Aqua viability dye (Fixable Viability Kit) to discriminate between live and dead cells, and Fc Block (Biolegend) was applied to reduce nonspecific antibody interactions. E7-specific CD8^+^ T cells within the PBMCs were detected by staining with PE-labeled E7 tetramer (1:200) and APC-A750-conjugated anti-mouse CD8α antibody (1:1000) for 30 min at 4 °C. MDSCs were identified by staining with APC-conjugated anti-mouse CD11b (1:1000), PE-conjugated anti-mouse Ly6G (1:1000), and PerCP/Cy5.5-conjugated anti-mouse Ly6C (1:1000) under the same conditions. After washing, samples were run on a Beckman Coulter CytoFLEX S flow cytometer (Beckman Coulter Life Sciences, Indianapolis, IN, USA), and data were processed using FlowJo version 10.4 (FlowJo LLC, Ashland, OR, USA).

### 4.7. Detection of Anti-Spike Antibodies

The 2 × 10^5^ Spike-expressing cells were seeded in 96-well plates and washed. Cells were then incubated with 100 µL of mouse serum (1:500 diluted) for 30 min at 4 °C. After washing, PE-conjugated anti-IgG (1:1000) was added to detect serum anti-spike antibodies and incubated for 30 min at 4 °C. Following a final wash, samples were analyzed on a Beckman Coulter CytoFLEX S flow cytometer, and data were processed using FlowJo (version 10.4, FlowJo LLC).

### 4.8. In Vivo Tumor Protection and Treatment Experiments

For prophylactic studies, C57BL/6 mice (*n* = 5 per group) were immunized intramuscularly with 2 µg of DNA followed by electroporation using one of the following constructs: (1) pcDNA3-E7(49-57), (2) pcDNA3-S-E7(49-57), or (3) pcDNA3-S-RE7(49-57). A booster immunization was administered one week later. Two weeks after the booster vaccination, mice were subcutaneously challenged with 1 × 10^5^ TC-1 cells in 100 µL HBSS using a 30-gauge needle at the left flank. A control group of untreated tumor-challenged mice was included. Survival was monitored for 60 days after tumor challenge. To assess long-term protection, surviving mice were rechallenged with 1 × 10^5^ TC-1 cells 11 weeks after the initial tumor challenge, and survival was monitored for an additional 90 days following tumor rechallenge.

For therapeutic studies, C57BL/6 mice (*n* = 5 per group) were subcutaneously challenged with 1 × 10^5^ TC-1 cells in 100 µL HBSS using a 30-gauge needle in the left flank. Three days after tumor challenge, mice received a 10 µg intramuscular prime immunization with one of the following plasmids: (1) pcDNA3-E7(49-57), (2) pcDNA3-S-E7(49-57), or (3) pcDNA3-S-RE7(49-57), followed by electroporation. Booster immunizations using the same regimen were given at one and two weeks after the initial immunization. Survival was monitored for 120 days following tumor implantation.

Tumor development was assessed weekly by palpation after tumor challenge, and tumor length and width were measured using a digital caliper. Tumor volume (mm^3^) was calculated using the formula: length × length × width × 0.5. Mice were euthanized when tumor diameter exceeded 17 mm or when other humane endpoints defined by the Johns Hopkins Animal Care and Use Committee were reached, at which point they were considered to have succumbed to tumor burden.

### 4.9. Statistical Analysis

All data are displayed as mean  ±  standard deviation (S.D). Kaplan–Meier survival curves were generated to evaluate survival rates and log-rank tests were performed to compare survival time between treatment groups. The statistical significance is determined by ordinary one-way ANOVA with Tukey–Kramer multiple comparison, two-way ANOVA with the Geisser–Greenhouse correction followed by a Tukey post test using the GraphPad Prism 10 software (GraphPad, Boston, MA, USA). All *p*  ≤  0.05 were considered significant (* *p*  <  0.05; ** *p*  <  0.01; *** *p*  <  0.001; **** *p*  <  0.0001).

## Data Availability

The original contributions presented in this study are included in the article/Supplementary Material. Further inquiries can be directed to the corresponding authors.

## Supplementary Materials

The following supporting information can be downloaded at: https://www.mdpi.com/article/10.3390/ijms27146249/s1.
