# Supplementary material for: A Spike-Linked HPV16 E7 DNA Vaccine Induces Potent Antitumor and Anti-Spike Immune Responses
Source: Int J Mol Sci. 2026 Jul 14;27(14):6249. doi: 10.3390/ijms27146249 (PMC13410571; doi:10.3390/ijms27146249)
Supplement: Supplementary file 1 [file ijms-27-06249-s001.zip › ijms-4308150-supplementary.pdf]

Supplementary Figure

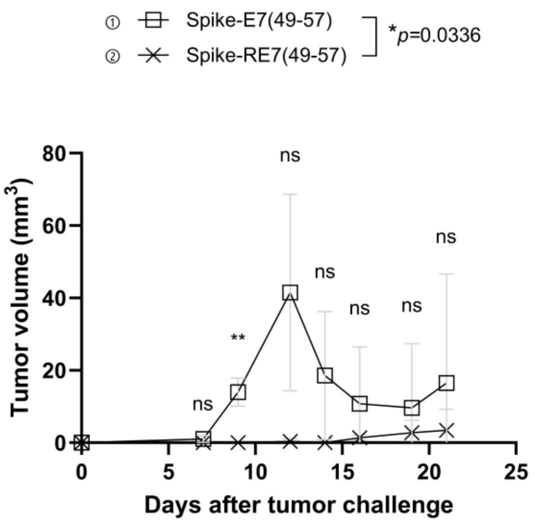

Figure S1. Zoomed line graph of Fig. 5b illustrating tumor volume progression in TC-1 tumor-challenged mice (Control and E7(49-57)-treated groups are not shown). Data are presented as mean ± SD and analyzed using one-way ANOVA (c). *p < .05, **p < .01, ns: not significant.

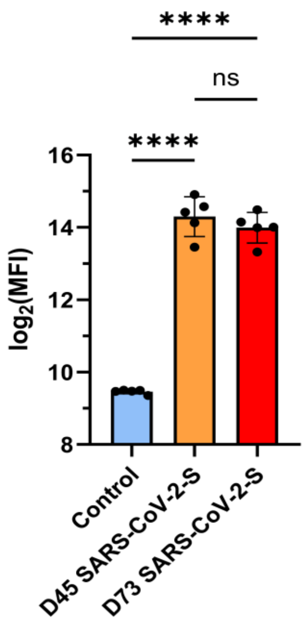

Figure S2. On day 1, mice were immunized intramuscularly with 10 µg of pCMV3-SARS-CoV-2-S, followed by electroporation. On days 8 and 15, the mice were boosted using the same regimen. On day 45, sera were collected from mice, and anti-spike antibody levels were analyzed by flow cytometry. Bar graph summarizing flow cytometry results, with the y-axis representing the $\log_2$ of median fluorescence intensity (MFI). Data are presented as mean ± SD and analyzed using one-way ANOVA with Tukey post test, ****p < .01, ns: not significant.
